# Neurocysticercosis presenting with oculomotor nerve palsy: Case report and literature review

**DOI:** 10.1016/j.idcr.2023.e01788

**Published:** 2023-05-06

**Authors:** Almurtada Razok, Maisa Ali, Abdullah Shams, Muhammad Zahid

**Affiliations:** a Department of Internal Medicine, John H. Stroger Jr. Hospital of Cook County, Chicago, IL 60612, USA; b Department of Infectious Diseases, Hamad Medical Corporation, Doha PO 3050, Qatar; c Department of Cardiology, Hamad Medical Corporation, Doha PO 3050, Qatar; d Department of Internal Medicine, Hamad Medical Corporation, Doha PO 3050, Qatar

**Keywords:** Neurocysticercosis, Taenia solium, Oculomotor nerve, Ptosis, Qatar

## Abstract

Neurocysticercosis (NCC), a central nervous system infection caused by the cystic larvae of Taenia Solium, is endemic in many low-to-middle income countries. NCC is known to have a variety of presentations depending on the size and site of involvement including chronic headaches, seizures, hydrocephalus, and ischemic insults. NCC has also been rarely associated with cranial nerve palsies. We report the case of a 26-year-old Nepalese lady who presented with isolated left-sided oculomotor nerve palsy and was found to have midbrain NCC. She was treated with anthelminthic agents and corticosteroids which led to clinical improvement. NCC can present with a variety of focal neurological syndromes. To the best of our knowledge, this is the first case report of NCC presenting with third cranial nerve palsy in the state of Qatar and the middle east. We also review the literature for other cases of NCC which presented with isolated oculomotor nerve palsy.

## Introduction

Taenia Solium or the pork tapeworm causes human cysticercosis, which is considered one of the zoonotic, endemic diseases in many countries of Latin America, South Asia, and Africa. T. Solium can infect the Subcutaneous tissue, muscles, and orbital structures. Neurocysticercosis (NCC), an infection of the central nervous system (CNS) by the larval form of the parasite, is responsible for at least one-third of acquired epilepsy cases in many of the endemic countries [1]. Adult-onset seizure is one of the common clinical presentations of NCC alongside headaches, focal neurological deficits, psychiatric manifestations, and intracranial hypertension caused mainly by hydrocephalus. Recent epidemiological research suggested that the increasing prevalence of adult-onset epilepsy in Latin America can be attributed to the growing incidence of NCC [2]. NCC is mainly acquired through ingesting undercooked pork and fecal-oral transmission from other carriers. Risk factors for acquiring NCC include under-regulation of pig farms, compromised water and food safety and poor sanitary conditions. In the gulf states, NCC is not considered an endemic disease, however due to the high influx of immigrants from countries where NCC is endemic, it has been acquiring increasing attention in the recent years. Between 2003 and 2011, 39 cases of NCC were reported in the Arabic Peninsula including Saudi Arabia, Kuwait, and Qatar. Seizure was the most frequent presentation, occurring in 90 % of patients while 5 % had focal neurological deficits, 62 % were less than 18 years of age and 64 % were females [3]. Four cases of NCC were reported in the state of Qatar in a 10-year period, however large-scale epidemiological studies assessing the prevalence and risk factors of NCC are lacking [4].

## Case presentation

**Fig. 1:**
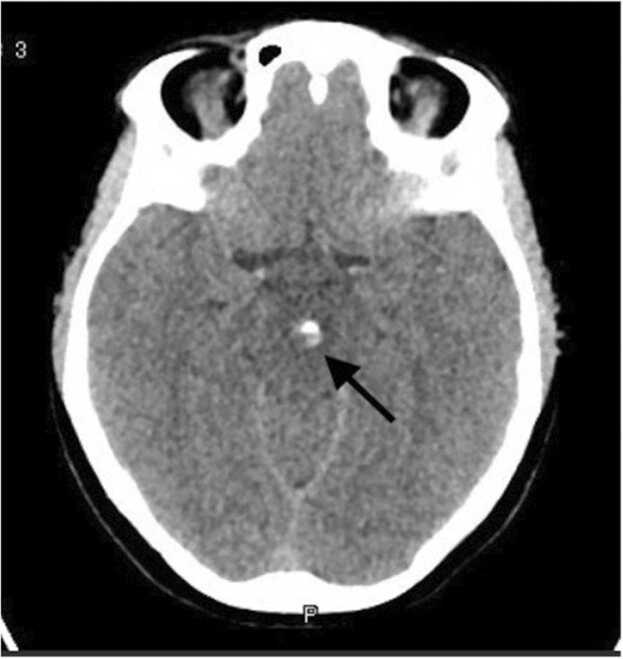
Cross-sectional view of CT brain showing a focal hyperdense calcified lesion measuring 6 mm in the midline portion of the midbrain (black arrow).

**Fig. 2:**
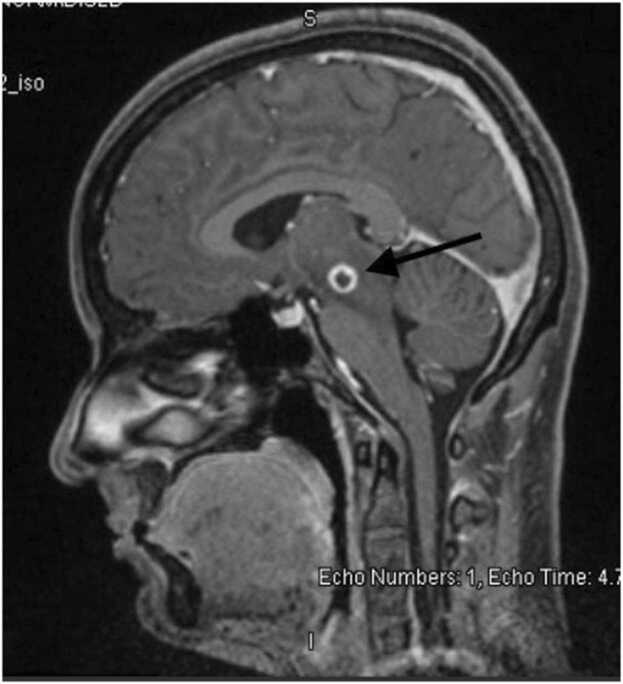
Sagittal view of MRI brain showing a midbrain ring-enhancing lesion 1.2 × 0.9 × 1 cm with central nodular calcification and significant peri-lesional edema (black arrow).

A 26-year-old Nepalese lady with no previous medical history presented with chief complaints of headache and left-sided monocular blurry vision for one week duration. The headache was located in the occipital region and was intermittent with no aggravating or relieving factors. There was no fever, nausea, vomiting or photophobia. She denied any sick contacts, animal exposure, history of recent infections, night sweats, weight loss, loss of appetite or change in bowel habits. Family history was negative, and her last international travel was to Nepal four months prior to presentation. She was married, had one child, and worked in catering department. Of note, there was no handling of pork meat as part of her job. Vital signs were normal and cranial nerve examination revealed left-sided ptosis and dilated left pupil with sluggish reaction to light. There was mild impairment in adduction, however other extraocular movements were preserved. There were no sensory or motor deficits in any of the extremities and meningeal signs were absent. Rest of the systemic examination was normal. Laboratory workup revealed normal complete blood count, renal and liver function tests. Her inflammatory markers including C-reactive protein and ESR were also normal. Computed tomography (CT) scan of the brain showed a focal hyperdense calcified lesion measuring 6 mm in the midbrain and a calcified focal lesion measuring 5 mm in the left frontal lobe. There was no midline shift or hydrocephalus, and the lesions were interpreted as calcified granulomas (Fig. 1). Magnetic resonance imaging (MRI) of the brain showed a midbrain ring-enhancing lesion with central nodular calcification and significant peri-lesional edema. Another ring-enhancing nodular calcified lesion without edema was reported in the left parasagittal frontal lobe giving an overall impression of granulomatous disease consistent with NCC (Fig. 2). Due to these findings, enzyme-linked immunoelectrotransfer blot (EITB) for anticysticercal antibodies was sent and was reportedly positive. Neurology and infectious disease teams were consulted, and the patient was started on a tapering regimen of dexamethasone for 14 days, in addition to albendazole and praziquantel. The patient was discharged four days later after improvement in ptosis and blurry vision. Unfortunately, post-discharge follow up was not established as the patient travelled back to her home country.

## Discussion

NCC can manifest in various clinical presentations depending on the host immune response to parasitic antigens, the number and location of lesions, and the degree of associated peri-lesional inflammation and edema. NCC lesions can occur in brain parenchyma or in the subarachnoid space and ventricles, referred to as extra-parenchymal NCC. Parenchymal NCC can lead to the formation of confined intraparenchymal viable cysts, solitary enhancing nodules or widespread massive cyst formation which in some instances can reach up to hundreds in the same patient. Parenchymal NCC can affect the cerebral hemispheres, basal ganglia, cerebellum, and the midbrain. Midbrain involvement can occur in isolation or more commonly, in the context of disseminated brain cysticercosis. Isolated brainstem NCC is one of the rare manifestations and only 29 cases in a review performed in 2013 were detected in the published literature [5]. We review the literature for five previously reported cases of isolated oculomotor nerve palsy due to NCC. The first case was reported in 2004 in a 34-year-old gentleman with bilateral 3rd nerve palsy with a relapse-remit course. He had bilateral ptosis, complete on the left and partial on the right with pupillary involvement on the left. Magnetic resonance imaging (MRI) of the brain showed a solitary ring-enhancing lesion in the tegmentum of the left midbrain. After treatment with corticosteroids, his symptoms resolved, and repeat neuroimaging showed regression of the midbrain lesion at four months follow-up [6]. The second case was also reported in 2004 in a 59-year-old gentleman who presented with acute-onset headache and diplopia. He had impaired adduction and elevation of the left eye with pupillary involvement. MRI brain showed ring-enhancing lesions in the tegmentum of the left midbrain and occipital pole. The patient was started on praziquantel for 15 days and intravenous methylprednisolone for six days, after which he had a resolution of his complaints. Due to symptoms recurrence, an additional course of IV methylprednisolone and dexamethasone was administered, and a slower taper of the prednisolone dose was provided [7]. The third case was reported in 2006 in a 25-year-old lady who presented with acute headache and partial, pupil-sparing left-sided ptosis. She was found to have palsy of the superior division of the left 3rd cranial nerve and neuroimaging showed a single ring-enhancing lesion in the left midbrain with peri-lesional edema. The diagnosis was confirmed with serum enzyme-linked immunosorbent assay (ELISA). Interestingly, this case was only the third case of superior divisional oculomotor nerve palsy caused by intrinsic brainstem disease, after multiple sclerosis and stroke. She was treated with albendazole, after which her complaints significantly improved [8]. The fourth case was reported in 2011 in a 16-year-old gentleman who presented with right sided ptosis and diplopia for one week. Physical Exam showed impaired adduction and elevation of the right eye with dilated pupil and impaired response to light. MRI of the brain showed a single ring-enhancing lesion in the right midbrain. The patient responded to albendazole and dexamethasone within three weeks [9]. The final case was also reported in 2011 in a 25-year-old gentleman who presented with acute-onset left-sided ptosis and diplopia for five days. Physical exam was consistent with pupil-sparing left sided 3rd cranial nerve palsy and brain MRI showed a solitary ring-enhancing lesion in the left midbrain. Both serum and CSF ELISA were positive. He was treated with steroids which led to clinical and radiological improvement in one and three months, respectively [10]. The mean age of reported cases was 31.8 years with all cases reported below 60 years of age. (4/5 cases) or 80 % of patients were males. There is a preponderance for the left side as it was involved in (4/5 cases) or 80 %, which our patient had as well. The pupil was affected in 60 % or 3/5 of the cases, which our patient also had. Four out of the five cases described above had a single ring-enhancing lesion involving the midbrain. Brain imaging in our patient showed two ring-enhancing lesions in the midbrain and frontal lobe. 80 % or 4/5 of cases received corticosteroids and 60 % or 3/5 of cases received anti-helminthic therapy either albendazole or praziquantel. 100 % of the cases demonstrated a satisfactory response to the prescribed treatment. Our patient was treated with dexamethasone, albendazole and praziquantel which led to a fast improvement of the presenting symptoms within three days. We summarize the main features of the previously reported cases in Table 1. Diagnostic criteria of NCC include absolute, major, and minor. These criteria include histopathologic, serologic, radiographic, epidemiologic, and clinical findings [11]. Our patient met one major radiographic, one major serologic, one minor clinical and one epidemiologic criterion.

**Table 1:** Summary of the previously reported cases of NCC presenting with oculomotor nerve palsy.

Author	Age	Gender	Laterality of 3rd nerve palsy	Pupil	Number of ring-enhancing lesions	Steroids
Mokta J et al. 2004 [6]	34 years	Male	Bilateral	Involved	Single	Yes
Kim JS et al. 2004 [7]	59 years	Male	Left	Involved	Two	Yes
Chotmongkol V et al. 2006 [8]	25 years	Female	Left	Spared	Single	No
Ranjith MP et al. 2011 [9]	16 years	Male	Right	Involved	Single	Yes
Khurana N et al. 2011 [10]	25 years	Male	Left	Spared	Single	Yes

## Conclusions

Oculomotor nerve palsy is one of the most common cranial nerve palsies and can occur due to various etiologies including mass lesions, ischemic injury, or demyelinating disease. As the nucleus is located at the level of the superior colliculus in the midbrain, virtually any pathology including that area, can lead to 3rd cranial nerve palsy. NCC is one of the parasitic infections that occurs in both endemic and non-endemic regions and can involve a wide array of CNS locations. NCC of the midbrain leading to isolated oculomotor nerve palsy has been rarely reported; therefore, we present this case to be added to the existing literature with hopes that it will contribute to larger-scale studies in the future. Understanding the epidemiology of midbrain NCC can help better understand its pathophysiology, clinical manifestations, and prognosis.

## Ethical approval

Not applicable, this manuscript is a case report.

## Source of funding

This article did not receive any specific grants from funding agencies in the public, commercial, or not-for-profit sectors.

## Conflicts of interest

None to be declared.
